# Deciphering the biocontrol strategies of *Trichoderma yunnanense* TM10 against *Fusarium oxysporum* f. sp. *cepae,* the causal agent of wilt disease in shallots (*Allium cepa* var. *aggregatum*)

**DOI:** 10.1016/j.crmicr.2025.100484

**Published:** 2025-10-03

**Authors:** Muhammad Adil Awal, Dedat Prismantoro, Reza Fauzi Dwisandi, Kah-Ooi Chua, Muhamad Shakirin Mispan, Nurul Shamsinah Mohd Suhaimi, Ratu Safitri, Febri Doni

**Affiliations:** a Department of Biology, Faculty of Mathematics and Natural Sciences, Universitas Padjadjaran, Jatinangor, 45363, West Java, Indonesia; b Doctorate Program in Biotechnology, Graduate School, Universitas Padjadjaran, Bandung, 40132, West Java, Indonesia; c Institute of Biological Sciences, Faculty of Science, Universiti Malaya, Kuala Lumpur, 50603, Malaysia; d Centre for Research in Biotechnology for Agriculture (CEBAR), Universiti Malaya, Kuala Lumpur, 50603, Malaysia; e Department of Global Development, Cornell University, Ithaca, NY, 14853, USA

**Keywords:** Biocontrol, *Fusarium oxysporum*, *Fusarium* wilt, shallots, *Trichoderma yunnanense*

## Abstract

The fungal genus *Trichoderma* has demonstrated significant potential for agricultural applications, particularly as a plant growth promoter and biocontrol agent against various phytopathogens. This study evaluated *Trichoderma yunnanense* TM10 as a biocontrol agent against *Fusarium oxysporum* f. sp. *cepae* (Foc) in shallots through both *in vitro* and *in vivo* assays. *In vitro* assays demonstrated that *T. yunnanense* TM10 effectively inhibited the growth of Foc through several mechanisms, including mycoparasitism, antibiosis, and competition for space between TM10 and Foc. Gas chromatography-mass spectrometry analysis identified eight volatile organic compounds exhibiting antimicrobial potential, while liquid chromatography-mass spectrometry analysis revealed eighteen non-volatile organic compounds with antifungal properties against Foc. *In vivo* assays showed that TM10 reduced disease severity by approximately 53.5 % and improved plant physiological characteristics, as shown by increased chlorophyll content, carotenoids, phenolic content, and antioxidant enzyme activity. Morphological improvements were also evident, with greater root and leaf elongation, higher biomass accumulation, and a higher number of leaves compared to pathogen-infected control plants. These findings suggest that *T. yunnanense* TM10 controls Foc through multiple mechanisms while promoting plant health, making it a promising and sustainable biocontrol agent for managing *Fusarium* wilt in shallots.

## Highlights

• *T. yunnanense* TM10 inhibits *Fusarium* via mycoparasitism, antibiosis, and competition.
• Eight antimicrobial VOCs and 18 antifungal non-VOCs were identified from *T. yunnanense* TM10.
• *T. yunnanense* TM10 TM10 reduced *Fusarium* disease severity in shallots by 53.5 %.
• *T. yunnanense* TM10 increased chlorophyll, phenolics, and antioxidants in shallot plants.
• *T. yunnanense* TM10 enhanced root length, leaf growth, and biomass in shallot plants.

## Graphical abstract

**Figure:**
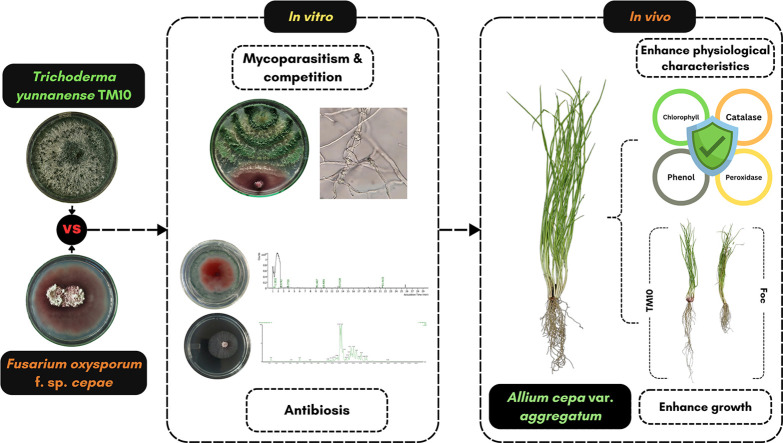

## 1 Introduction

Shallots (*Allium cepa* var. *aggregatum*) are an important horticultural crop, with global production reaching approximately 5.07 million metric tons in 2023 (Statista, 2025). This crop plant is a vital commodity. It is rich in fiber, folic acid, vitamin B6, magnesium, calcium, potassium, phosphorus, various other vitamins and minerals, and abundant in bioactive compounds (Chakraborty et al., 2022; Stoica et al., 2023). However, shallot cultivation often faces various challenges, including poor soil fertility, inadequate production techniques, unimproved varieties, limited availability of good quality seeds, and difficulties in controlling pests and diseases (Sharifi-Rad et al., 2016; Wenli et al., 2019; Degani et al., 2022; Hamidou et al., 2022; Yeshiwas et al., 2023). The primary phytopathogens attacking shallot plants are fungal pathogens, which can lead to wilting and even plant death, thus significantly impacting shallot production (Hamidou et al., 2022).

Fungal pathogens constitute the most serious constraint to shallot cultivation (Hamidou et al., 2022). Major fungal genera involved in shallot diseases include *Colletotrichum* spp. (anthracnose), *Botrytis* spp. (leaf blight), *Phoma* spp. (pink root), *Alternaria* spp. (leaf blight), and *Fusarium oxysporum* f. sp. *cepae* (*Fusarium* wilt) (Dutta et al., 2022; Sultana and Hossain, 2021; Steentjes et al., 2021; Htun et al., 2022; Degani et al., 2022). Among these, *F. oxysporum* f. sp. *cepae* (Foc) is particularly destructive due to its high virulence and specificity toward shallots and other members of the *Allium* genus (Bektaş et al., 2019; Nasr, 2018; Maulidha et al., 2024). Foc is also known to produce phytotoxic compounds, including mycotoxins, which disrupt the integrity and flexibility of the plasma membrane in shallot leaf cells (Perincherry et al., 2019; Kalman et al., 2020). These disruptions contribute to characteristic symptoms such as foliar wilting and bulb rot, which severely impair shallot vitality and yield.

Synthetic fungicides are commonly applied to manage infections caused by Foc, but their overall effectiveness is often limited. Chemical compounds such as thiophanate methyl, carbendazim, pyrimethanil, fludioxonil, azoxystrobin, prochloraz, tebuconazole, pyraclostorobin-boscalid, fludioxonil-sedaxane, and azoxystrobin-tebuconazole have been reported to suppress the growth of Foc infections, but their overall efficacy remains limited (Degani and Kalman, 2021; Bektaş et al., 2019; Panno et al., 2021). Moreover, the extensive use of these fungicides has led to various negative consequences, including environmental contamination, depletion of soil organic matter, and the emergence of resistant strains of plant pathogens (Feng et al., 2020; Gikas et al., 2022; Guo et al., 2021). These limitations highlight the need for safer and more sustainable disease management strategies.

Biological control using beneficial microorganisms has emerged as a promising alternative for sustainable crop protection (Sarrocco et al., 2023). Compared to chemical approach, biocontrol is considered environmentally friendly, as it reduces the risk of resistance development, and is able to enhance plant health and productivity (Thambugala et al., 2020; Palmieri et al., 2022). The fungal genus *Trichoderma* is one of the most widely studied groups of biological control agents (Dwisandi et al., 2024). *Trichoderma* can suppress numerous phytopathogens through mechanisms such as mycoparasitism, production of antibiotic-like compounds, competition for nutrients and space, and stimulation of plant defense responses (Guzmán-Guzmán et al., 2023; Awal et al., 2024; Prismantoro et al., 2024a). Recent studies further highlight their ability to improve soil health, regulate rhizosphere microbiota, and enhance crop tolerance to both biotic and abiotic stresses (Asghar et al., 2024; Awal et al., 2024).

More than 400 species of *Trichoderma* have been identified to date (Wijayawardene et al., 2020), but only a few species such as *T. harzianum, T. viride, T. asperellum, T. hamatum*, and *T. koningiopsis* have been thoroughly investigated for their effectiveness against Foc. These species have shown inhibition rates ranging from 57 % to 82 % (Rajamohan et al., 2019; Moka et al., 2021). However, increasing evidence suggests that less-studied species may also hold significant potential. For instance, *T. yunnanense*, first described in 2007, has demonstrated antagonistic activity against a broad spectrum of pathogens including *F. oxysporum, F. solani, F. proliferatum, Phytophthora capsici, Aspergillus flavus, Rhizoctonia solani, Pyricularia oryzae*, and *C. gloeosporioides*, with inhibition levels reported between 61 % and 100 % (Yu et al., 2007; Yazid et al., 2023; Nguyen et al., 2023; Kumari and Sharfuddin, 2023; Prismantoro et al., 2025). Despite these promising results, the biocontrol capabilities of *T. yunnanense* remains relatively underexplored compared to other *Trichoderma* species (Zanfaño et al., 2024; Ferreira and Musumeci, 2021).

Recently we have successfully isolated a plant growth-promoting *T. yunnanense* TM10, a novel symbiotic fungus that has the capability to enhance rice growth, and control rice diseases through strong enzymatic and antagonistic activities (Akbari et al., 2024; Prismantoro et al., 2024b, 2025). However, information regarding the biocontrol capabilities of *T. yunnanense* TM10 against Foc infecting shallots has not yet been investigated. Given the substantial economic losses attributed to this pathogen, exploring novel biological control agents is of considerable agronomic importance. Therefore, this research aims to elucidate the biocontrol potential and effectiveness of *T. yunnanense* TM10 for controlling Foc, the causal agent of *Fusarium* wilt in shallots.

## 2 Material and methods

### 2.1 Fungal strains and culture conditions

The antagonistic fungal isolate *T. yunnanense* TM10 used in this study was obtained from the Applied Microbiology Laboratory, Faculty of Mathematics and Natural Sciences, Universitas Padjadjaran, Indonesia (Akbari et al., 2024; Prismantoro et al., 2024b, 2025). The pathogenic fungal isolate *F. oxysporum* f. sp. *cepae* (Foc) was obtained from the Plant Protection Biotechnology Laboratory, Faculty of Agriculture, Universitas Padjadjaran, Indonesia (Prabowo et al., 2020; Suganda et al., 2019). The fungal strains were grown on potato dextrose agar (PDA), incubated at 25±2 °C for 5-7 days, and stored at 4 °C for further experiments.

### 2.2 Morphological characterization

Macroscopic and microscopic observations were conducted to characterize *T. yunnanense* TM10 and Foc. For macroscopic analysis, each fungal isolate was cultured on PDA and incubated at 28 °C to 30 °C. The *T. yunnanense* TM10 isolate was incubated for 7 days, while the Foc isolate was incubated for 10 days to assess colony growth and appearance. Microscopic characterization was carried out using the moist chamber method, with incubation at 28 °C to 30 °C for 3 to 4 days to promote the development of reproductive structures. Fungal structures such as conidia and hyphae were examined under a light microscope (Olympus CX21, Tokyo, Japan).

### 2.3 Evaluation of the antagonistic activity of *T. yunnanense* TM10 under *in vitro* condition

#### 2.3.1 Dual culture assay

The antagonistic activity of *T. yunnanense* TM10 against Foc was first evaluated using the dual culture method. A 5 mm agar plug from each fungal isolate was placed 2 cm from the edge of a Petri dish containing PDA, maintaining an approximate distance of 4.5 cm between the two colonies (Bunbury-Blanchette and Walker, 2019). *T. yunnanense* TM10 was inoculated two days after the inoculation of Foc (Sandle, 2014; Ghanbarzadeh et al., 2016; Olowe et al., 2022). Radial growth of both fungi was measured every 24 h for seven days. The inhibitory effect of *T. yunnanense* TM10 was quantified using the percentage inhibition of radial growth (PIRG) below:

$$PIRG\;\left(\%\right)=\;\frac{Df-Dt\;}{Df}\;x\;100$$

Note:

Df = Diameter of Foc mycelium in control

Dt = Diameter of Foc mycelium in dual culture assay

#### 2.3.2 Culture filtrate assay

The culture filtrate assay was adapted from Boukaew et al. (2024) and Naglot et al. (2015), with minor modifications. *T. yunnanense* TM10 was cultured in potato dextrose broth (PDB) and incubated in a shaker at room temperature for 14 days. Samples were collected at two-day intervals (days 0, 2, 4, 6, 8, 10, 12, and 14) and filtered through a Whatman No. 44 filter paper. The filtrate was centrifuged at 4,000 rpm for 15 mins, and the supernatant was further sterilized using a 0.22 µm syringe filter. For the assay, 25 mL of *T. yunnanense* TM10 culture filtrate was mixed with 25 mL of molten PDA to create a 1:1 medium. A 5 mm agar plug of Foc was placed in the center of each plate and incubated at 28 °C to 30 °C. Control plates were prepared by growing Foc on PDA without filtrate. The diameter of the Foc colony was measured after incubation, and the results were compared between treatments and controls using the previously described PIRG formula.

#### 2.3.3 Volatile compound assay

The volatile compound-mediated antagonism of *T. yunnanense* TM10 against Foc was assessed using a sealed double-plate method, following Moutassem et al. (2020) and Chan et al. (2023), with slight modifications. A plug of 5 mm diameter from a 7-day-old *T. yunnanense* TM10 culture was inoculated onto PDA, and the lid of the Petri dish was replaced with the base of another dish containing a 7-day-old Foc culture. The two dishes were sealed together with parafilm to allow only volatile exchange, then incubated at 28 °C to 30 °C. The radial growth of Foc was measured on days 2, 4, 6, 8, 10, 12, and 14. The inhibitory effect was calculated using the previously described PIRG formula.

#### 2.3.4 Microscopic observation of antagonism by *T. yunnanense* TM10

To observe the microscopic antagonism, the interaction between *T. yunnanense* TM10 and Foc was examined using a modified slide culture method (Reddy et al., 2014). PDA medium was poured into sterile Petri dishes, allowed to solidify, and then cut into sections, which were transferred onto sterile glass slides for dual inoculation of both fungi on opposite sides. The slides were incubated at 28 °C for 3 days. The slides were observed under a light microscope at 400–1,000 times magnification for antagonistic structures such as hyphal coiling, penetration, and degradation.

#### 2.3.5 Volatile organic compounds profiling of *T. yunnanense* TM10

Volatile organic compounds (VOCs) produced by *T. yunnanense* TM10 during interaction with Foc were analyzed using headspace solid-phase microextraction (HS-SPME) and gas chromatography–mass spectrometry (GC–MS) (Mihaylova et al. 2022). The analysis was conducted at the Characterization Laboratory, National Research and Innovation Agency, Bogor, Indonesia. Foc-enriched medium was prepared based on method described by Javeria et al. (2020) and Deep et al. (2014) by culturing Foc in 500 mL PDB at 28–30 °C for 7 days, followed by centrifugation, washing, boiling, and freezing at –20 °C. *T. yunnanense* TM10 was inoculated into 50 mL of this medium in a 200 mL Erlenmeyer flask. A 5 mm agar plug of *T. yunnanense* TM10 was inoculated into the flask and incubated at 28 °C. On the 7th day, an HS-SPME fiber (50/30 µm DVB/PDMS) was inserted into the flask to collect VOCs.The collected VOCs were desorbed into a 5977B GC/MSD system at 220 °C for 1 min using an HP-5MS column, with helium as the carrier gas (1 mL/min). The oven temperature was programmed from 40 °C to 260 °C, and spectra were acquired in full scan mode (50–500 m/z). Compounds were identified by comparing the chromatographic peaks against the reference libraries.

#### 2.3.6 Non-volatile organic compounds profiling by *T. yunnanense* TM10

*T. yunnanense* TM10 was inoculated onto a minimal medium enriched with Foc hyphae, following the methods of Javeria et al. (2020) and Deep et al. (2014). The extraction of non-volatile metabolites was conducted with modifications based on Wang et al. (2022). Culture filtrates were filtered and extracted using an equal volume of methanol, then rotary evaporated under vacuum at 40 °C. The dried extracts were re-dissolved in methanol prior to analysis. All procedures were performed at the Indonesian National Police Forensic Laboratory Center, Bogor, West Java. Identification of the metabolite components was carried out using liquid chromatography–mass spectrometry (LC–MS).

Chromatographic separation was performed using an ACQUITY UPLC® HSS C18 column (1.8 µm, 2.1 × 100 mm; Waters, USA), with the column maintained at 50 °C and the room temperature at 25 °C. The mobile phase consisted of water with 5 mM ammonium formate (A) and acetonitrile with 0.05 % formic acid (B), operated at a flow rate of 0.2 mL/min in a step gradient for 23 mins. The injection volume was 5 µL, filtered through a 0.2 µm syringe filter prior to injection. Mass spectrometry analysis was conducted in positive ion mode using electrospray ionization (ESI), with a scanning range of 50–1200 m/z. The ion source and desolvation temperatures were set at 100 °C and 350 °C, respectively, with a cone gas flow of 0 L/h and a desolvation gas flow of 793 L/h. Collision energy was applied in a range of 4 to 60 eV. Compound identification was based on peak intensity and spectral data, processed using MassLynx version 4.1.

### 2.4 Evaluation of the antagonistic activity of *T. yunnanense* TM10 against *F. oxysporum* f. sp. *cepae* under *in vivo* conditions in *A. cepa* var. *aggregatum* plants

#### 2.4.1 Preparation of *T. yunnanense* TM10 and Foc conidial suspensions

Conidial suspensions of *T. yunnanense* TM10 and Foc were prepared at a concentration of 1 × 10⁷ conidia/mL. *T. yunnanense* TM10 isolate cultured on PDA medium for 7 days at 28 °C, were harvested by scraping the surface with 10 mL of sterile distilled water (Akbari et al., 2024). The resulting suspension was transferred to an Erlenmeyer flask and diluted with sterile water to the target concentration. Conidial counts were determined using a hemocytometer based on the method described by Anhar et al. (2018), using the following formula:

$$Conidia\;density=\frac{n\;\times \;5\;\times d}{Vh}$$

Note: n = Number of conidia, d = Dilution factor

Vh = Hemocytometer volume

Foc conidial suspensions were prepared similarly. Isolates grown on PDA for 7 days at 28 °C were harvested using 10 mL of sterile distilled water (Zhang et al., 2023; Poromarto et al., 2022). The suspension was adjusted to a final concentration of 1 × 10⁷ conidia/mL through dilution and quantified using the same hemocytometer method as described above.

#### 2.4.2 *In vivo* experiment

The *in planta* assay was carried out under greenhouse conditions at the Plant Experimentation Station, Department of Biology, Universitas Padjadjaran. The procedures were adapted and modified from Rivera-Méndez et al. (2019), Zapata-Sarmiento et al. (2020), and Poromarto et al. (2022).

Healthy shallots bulbs (3-4 cm in diameter) were directy planted into 25 × 25 cm polybags. Each polybag was filled with 4 kg of a sterilized soil:peat:manure mixture (2:1:1), previously autoclaved at 121 °C for 60 mins. Plant were watered daily with sterile water to maintain adequate soil moisture. After three weeks of growth, treatments were arranged in a completely randomized design with ten replicates each: (i) Control, (ii) *T. yunnanense* TM10, (iii) Foc, and (iv) *T. yunnanense* TM10 + Foc. For treatments (ii) and (iv), *T. yunnanense* TM10, a spore suspension of *T. yunnanense* TM10 was prepared from 7-day-old PDA cultures, harvested with sterile distilled water, and adjusted to 1 × 10⁷ CFU/mL using a hemocytometer. Each plant was then treated by drenching the soil with 10 mL of this suspension.

For pathogen treatments (iii and iv), Foc inoculum was prepared in the same way and introduced at 1 × 10⁷ CFU/mL by drenching the root zone with 10 mL of the inoculum three days before TM10 application to ensure pathogen establishment. During this initial incubation, plants were covered with transparent plastic bags to maintain high humidity and promote infection. Two days after Foc inoculation, plastic covers were removed, and the plants were maintained under greenhouse conditions with natural sunlight. Environmental conditions were monitored throughout the experiment, with average temperature 30±4 °C, light intensity 320 ± 3 μmol m⁻² s⁻¹, relative humidity 80±3 %, and a natural photoperiod of 11 h 11 min ± 9 s over two growing seasons.

#### 2.4.3 Analysis of disease severity index

Disease severity was assessed using the disease severity index (DSI) based on methods described by Chiang et al. (2017), Zapata-Sarmiento et al. (2020), and Poromarto et al. (2022). Disease symptoms were evaluated at 14 days after inoculation using a 0–4 scale based on the extent of leaf yellowing and drying: 0 = no symptoms; 1 = 1-25 % yellowing and drying of leaves; 2 = 26-50 % yellowing and drying of leaves; 3 = more than 51-75 % yellowing and drying of leaves; and 4 = 76-100 % yellowing and drying of leaves (all leaves). Once the disease severity scale data were obtained, the percentage of disease severity was calculated using the following formula, based on the method of Chiang et al. (2017) and Harish et al. (2007):

$$DSI\;\left(\%\right)=\;\frac{\Sigma \left(Cf\;\times \;Sf\right)}{Tn\;\times M}\;X\;100$$

Note:

Cf = Class frequency

Sf = Score of rating class

Tn = Total number of observations

M = Maximum disease index

#### 2.4.4 Physiological analysis of shallot plants after treatments

Physiological responses of shallot plants were assessed after the *in vivo* assay, including total phenolic content, defense-related enzyme activity (catalase and peroxidase), and chlorophyll content.

##### 2.4.4.1 Total phenolic content

Total phenolics were analyzed based on Jaiswal et al. (2020). A 0.1 g leaf sample was homogenized in 10 mL of 70 % acetone and centrifuged. One milliliter of the supernatant was mixed with 1 mL of 1 N Folin–Ciocalteu reagent and 2 mL of 5 % sodium carbonate, and then diluted to a final volume of 10 mL with distilled water. The solution was heated until it turned blue and measured at 650 nm using a UV-Vis spectrophotometer. Gallic acid was used as the standard.

##### 2.4.4.2 Defense enzymes content

Catalase (CAT) and peroxidase (POD) activities were measured methods described by Panahandeh et al. (2016) and Pradhan et al. (2023). Freeze-dried leaf tissue was ground and stored at −80 °C. One gram of sample was extracted with 10 mL of 50 mM potassium phosphate buffer (pH 6.8), followed by centrifugation at 12,000 g for 20 mins at 4 °C. CAT activity: 50 µL of enzyme extract was mixed with 500 µL of 10 mM H₂O₂ and 2,450 µL of buffer, and absorbance was measured at 240 nm for 60 s. POD activity: 200 µL of extract was combined with 1,000 µL of 100 mM potassium phosphate buffer, 500 µL of 20 mM guaiacol, and 300 µL of 10 mM H₂O₂. Absorbance at 470 nm was recorded every 5 s for 90 s.

##### 2.4.4.3 Chlorophyll content

Chlorophyll *a*, chlorophyll *b*, and total chlorophyll were determined following Zapata-Sarmiento et al. (2020) and Sarangi et al. (2021) with slight modifications. A 0.1 g leaf sample was soaked in 10 mL of 95 % ethanol in the dark at 25 °C for 24 h. After centrifugation, the supernatant was analyzed spectrophotometrically at 645 and 663 nm. Chlorophyll concentrations were calculated using the following formulas:

$$\mathrm{Ch}l_{a}=12.7A_{663}\;-\;2.69A_{645}$$

$$\mathrm{Ch}l_{b}=22.9A_{645}\;-\;4.68A_{663}$$

$$Total\;Chlorophyll\;=\;Chl\;a\;+\;Chl\;b\;=\;20.2\;A_{645}\;+\;8.02\;A_{663}$$

$$\mathrm{Care}\mathrm{tono}\mathrm{ids}\;=\;\left(1000\;\times \;A_{470}\;-\;1.82\;\times \;\mathrm{Chl}\;a\;-\;85.02\;\times \;\mathrm{Chl}\;b\right)\;/\;198$$

#### 2.4.5 Morphological characteristics of shallot plants after treatments

Morphological observations were conducted based on research by Altintas and Bal (2008), with modification. Shallot plant morphology was measured after *in vivo* assay concluded, which was 10 days after Foc conidia application. The observed parameters included plant height (root length in cm and leaf length in cm), fresh weight (g), dry weight (g), and number of leaves (sheets). Each parameter was measured using calipers and an analytical balance.

### 2.5 Data analysis

All data were analyzed using SPSS software (version 26.0, IBM Corp., Armonk, NY, USA). Comparisons between two groups were conducted using Student’s t-test, while comparisons among more than two groups were first subjected to one-way analysis of variance (ANOVA) followed by Duncan’s Multiple Range Test (DMRT) for post hoc mean separation. Differences at P < 0.05 were considered statistically significant.

## 3 Results

### 3.1 Morphology of *T. yunnanense* TM10 and *F. oxysporum* f. sp. *cepae*

**Fig. 1:**
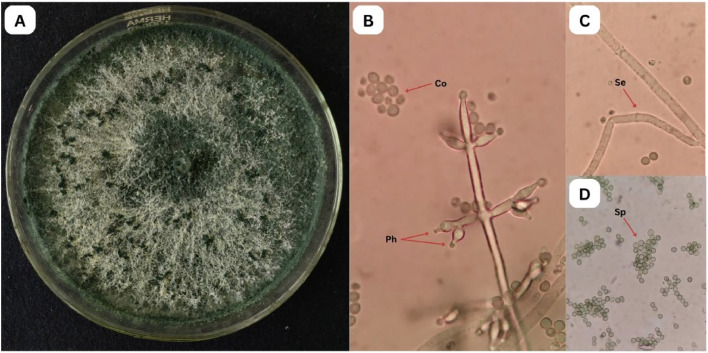
Morphological characteristics of *T. yunnanense* TM10. A. Macroscopic view; B-D. Microscopic view with 100-400X magnifications (Co=conidiospore, Ph=phialides, Se=septum, Sp=spore).

**Fig. 2:**
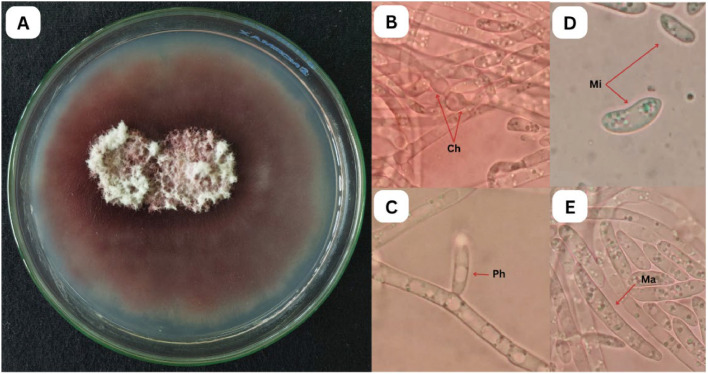
Morphological characteristics of Foc. A. Macroscopic view; B-E. Microscopic view with 100-400X magnifications (Ch=chlamydospore, Ph=phialides, Mi=Microconidia, Ma=Macroconidia).

The morphological characteristics of *T. yunnanense* TM10 and Foc were successfully observed through both macroscopic and microscopic examinations (Fig. 1, Fig. 2). Colonies of *T. yunnanense* TM10 initially appeared white (day 1 – 3), gradually turning light green after day 3, and becoming dark green by day 7, eventually covering the entire surface of the PDA. The colonies exhibited a cotton-like texture and emitted a coconut-like scent. Microscopic observation revealed that TM10 showed septate hyphae, lageniform to ampulliform phialides, and smooth-walled, oval to obovoid conidia ranging from light to dark green.

Foc colonies initially appeared white beneath the PDA medium at day 3, it then developed a pinkish-purple-brown color by day 5. By day 10, colonies fully covered the surface of PDA, exhibited a cotton-like texture in the center and no detectable odor. Microscopic observations revealed phialides, and three spore types: oval to kidney-shaped microconidia, sickle-shaped macroconidia, and thick-walled, round chlamydospores.

### 3.2 *In vitro* inhibition assay of *T. yunnanense* TM10 on *F. oxysporum* f. sp. *cepae*

Antagonistic activity of *T. yunnanense* TM10 against Foc was demonstrated through three *in vitro* assays: dual culture, culture filtrate, and volatile compound assays. Each assay resulted in varying degrees of inhibition, revealing that *T. yunnanense* TM10 exhibited different modes of antagonism. The inhibition values obtained from these assays are presented in Table 1 and described in detail in the following subsections.

**Table 1:** Comparison of each *in vitro* inhibition assay of *T. yunnanense* TM10 against Foc.

**Tests**	**Inhibition (%)**
Dual culture assay	76.90±1.04^c^
Culture filtrate assay	50.48±0.38^b^
Volatile compound assay	36.29±2.49^a^

Note: Mean values followed by the same lowercase letter within the same column indicate no significant difference according to DMRT test (*P* < 0.05). Data are presented as mean ± standard deviation.

#### 3.2.1 Dual culture assay

**Fig. 3:**
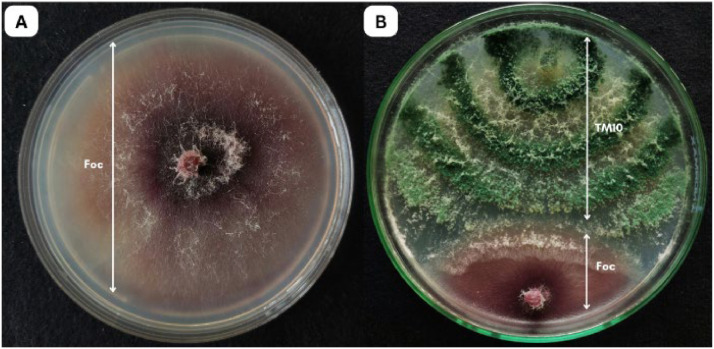
Dual culture assay showing the antagonistic activity of *T. yunnanense* TM10 against Foc: A. Control plate with Foc grown alone (day 10); B. Dual culture plate showing overgrowth of *T. yunnanense* TM10 against Foc (day 7 of dual culture assay).

In the dual culture assay, *T. yunnanense* TM10 exhibited strong antagonistic activity against Foc, despite being inoculated two days later. By day 5, *T. yunnanense* TM10 had reached the Foc colony and overgrown more than half of the PDA, indicating a rapid growth rate and highly competitive ability. The fungal interaction suggested effective competition for nutrient and space, which restricted the radial expansion of Foc. This overgrowth persisted through day 7, with *T. yunnanense* TM10 continuing to dominate the medium surface. The inhibition percentage recorded was 76.90±1.04 %, confirming its substantial biocontrol potential (Fig. 3).

#### 3.2.2 Culture filtrate assay

**Fig. 4:**
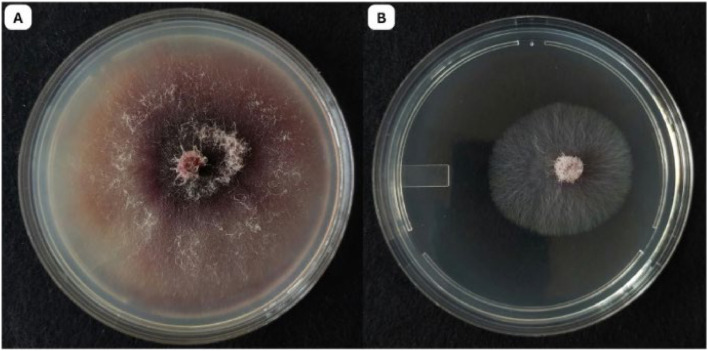
Culture filtrate assay showing the inhibitory effects of secondary metabolites produced by *T. yunnanense* TM10 against Foc. (A) Control plate with Foc grown alone and without culture filtrate of *T. yunnanense* TM10 and (B) Plate with culture filtrate of *T. yunnanense* TM10 (day 14) showing reduced growth of Foc.

The culture filtrate assay demonstrated that secondary metabolites produced by *T. yunnanense* TM10 were capable of inhibiting Foc growth (Fig. 4). Filtrates were collected at various intervals from cultures grown in potato dextrose broth enriched with autoclaved Foc hyphae and incorporated into PDA medium for Foc inoculation.

As shown in Table 2, the inhibition effect of *T. yunnanense* TM10 increased with filtrate age, with the highest suppression observed at 12 and 14 days, both yielding an inhibition rate of 50.48 % and the lowest Foc growth at 4.11 cm. In contrast, the 0-day filtrate exhibited minimal suppression (4.94±0.78 %), comparable to the control (0 %). These results indicate a time-dependent accumulation of antifungal compounds, with filtrate efficacy stabilizing after day 10, suggesting optimal metabolite production occurs between 10–14 days of incubation.

**Table 2:** Comparison of Foc growth in response to the age of *T. yunnanense* TM10 filtrate.

**Filtrate Age** (days)	**Growth of Foc** (cm)	**Inhibition Rate** (%)
Control	8.3±0.013	0^a^
0	7.89±0.064	4.94±0.78^b^
2	6.35±0.048	23.49±0.57^c^
4	5.77±0.037	30.48±0.44^d^
6	5.04±0.017	39.28±0.20^e^
8	4.87±0.013	41.33±0.15^e^
10	4.13±0.017	50.24±0.19^f^
12	4.11±0.020	50.48±0.24^f^
14	4.11±0.032	50.48±0.38^f^

Note: Mean values followed by the same lowercase letter within the same column indicate no significant difference according to DMRT test (*P* < 0.05). Data are presented as mean ± standard deviation.

#### 3.2.3 Volatile compound assay

**Fig. 5:**
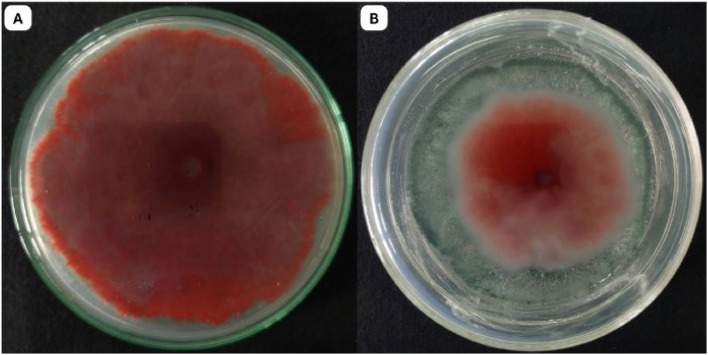
Comparison of Foc isolate between control plate (A) and Foc plate exposed to volatile compounds from *T. yunnanense* TM10 (Day 14) (B).

Volatile organic compounds (VOCs) produced by *T. yunnanense* TM10 inhibited the growth of Foc in a sealed double-plate setup. Compared to the control, Foc mycelial growth was significantly reduced in the presence of TM10 VOCs, reaching only 5.41 cm by day 10 compared to 8.5 cm in the control. The inhibition percentage was 36.29±2.49 %, indicating a moderate antibiosis effect mediated by volatile compounds (Fig. 5).

### 3.3 Observation of interaction between *T. yunnanense* TM10 and Foc

**Fig. 6:**
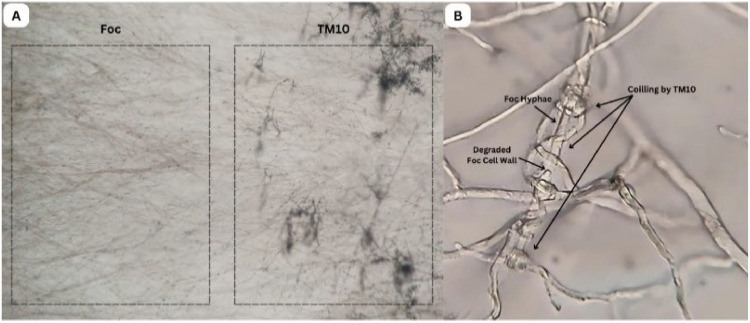
The interactions between *T. yunnanense* TM10 and Foc using the moist chamber method (A. 40X magnification and B. 1,000X magnification).

Microscopic observation using the slide culture method showed that *T. yunnanense* TM10 exhibited direct mycoparasitic activity against Foc. The TM10 hyphae were observed coiling around the Foc hyphae, a characteristic indicator of mycoparasitic interaction. This coiling was accompanied by visible signs of cell wall degradation in the Foc hyphae, suggesting structural damage. These results confirm that *T. yunnanense* TM10 engages in direct antagonism through physical interaction with Foc (Fig. 6).

### 3.4 Profiling and identification of antifungal metabolites from *T. yunnanense* TM10

**Fig. 7:**
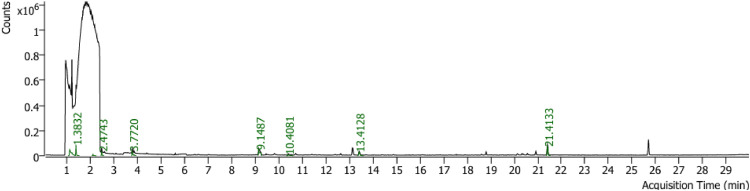
Spectrum of gas chromatography-mass spectrometry (GC-MS) from the ethyl acetate extract of *T. yunnanense* TM10.

GC-MS analysis identified volatile organic compounds (VOCs) produced by *T. yunnanense* TM10 grown on PDA medium enriched with autoclaved Foc hyphae (Fig. 7 and Table 3). Quantitative analysis based on peak area showed that ethanol was the dominant metabolite, detected at two retention times (1.12 and 1.38 min) and accounting for 25.78 % and 12.05 % of the total VOCs, respectively. Together, ethanol represented nearly 38 % of the volatile profile, consistent with its reported antimicrobial properties. Other major constituents included cyclotetrasiloxane, octamethyl- (17.48 %), which belongs to the siloxane group associated with plant protection activity, and butylated hydroxytoluene (BHT) (16.59 %), a substituted phenol known for both antioxidant and antimicrobial activities.

**Table 3:** List of volatile compounds successfully identified from gas chromatography-mass spectrometry (GC-MS) analysis.

**No**	**Retention Times**	**Compounds**	**Formulas**	**Component Areas**	**Area (%)**	**Molecular Weight (g/mol)**	**Biocontrol Activities**	**References**
1	1.12	Ethanol	C_2_H_6_O	281295.78	25.78	46.069	Antimicrobial	Steglińska et al. (2022); Medina-Romero et al. (2017); Kong et al. (2020)
2	1.38	Ethanol	C_2_H_6_O	131504.25	12.05	46.069	Antimicrobial	Steglińska et al. (2022); Medina-Romero et al. (2017); Kong et al. (2020)
3	2.10	Trichloromethane	CHCl_3_	19763.31	1.81	119.37	Antimicrobial	Thangaraj et al. (2020)
4	2.47	Butanal, 3-methyl-	C_5_H_10_O	60945.63	5.59	86.13	Antimicrobial	Jiang et al. (2021); Gao et al. (2018); Hung et al. (2013)
5	3.77	1-Butanol, 3-methyl-	C_5_H_12_O	101477.01	9.30	86.13	Antimicrobial	Jiang et al. (2021); Kong et al. (2020); Medina-Romero et al. (2017)Hung et al. (2013)
6	9.15	Cyclotetrasiloxane, octamethyl-	C_8_H_24_O_4_Si_4_	190685.19	17.48	356.7	Plant protection	Joo and Husein (2022); You et al., (2022); Braat et al. (2022); Ghasemi et al. (2021)
7	13.41	Naphthalene	C_10_H_8_	93485.89	8.57	128.17	Antimicrobial	Khan and Javaid (2021); Wang et al. (2011); Osman et al. (2023)
8	21.41	Butylated hydroxytoluene (BHT)	C_15_H_24_O	181026.89	16.59	220.35	Antioxidant, Antimicrobial	Sebestyen et al. (2022); Elshahawy and Marrez (2024); Kandasamy and Kandasamy (2014)

Additional compounds of notable abundance were 1-butanol, 3-methyl- (9.30 %), a tertiary alcohol with antimicrobial activity, and naphthalene (8.57 %), a polycyclic aromatic hydrocarbon with documented antifungal potential. VOCs present in moderate to low concentrations included butanal, 3-methyl- (5.59 %) and trichloromethane (1.81 %), which nevertheless contribute to the overall antifungal and antimicrobial activity spectrum of the extract. Overall, the VOC profile of *T. yunnanense* TM10 was dominated by ethanol, siloxanes, and phenolic antioxidants, collectively comprising more than 70 % of the total peak area, thereby providing strong support for its potential role in antifungal activity and plant protection.

**Fig. 8:**
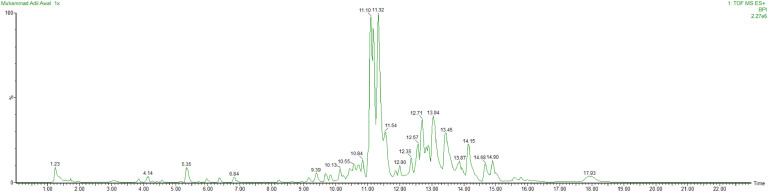
Spectrum of liquid chromatography-mass spectrometry (LC-MS) from *T. yunnanense* TM10.

LC-MS analysis of *T. yunnanense* TM10 culture extract identified a wide range of non-volatile metabolites with diverse chemical classifications (Fig. 8 and Table 4). Quantitative assessment based on relative peak area revealed that the extract was dominated by organic acid derivatives, particularly a malic acid–containing compound (RT 11.17; 32.39 % of total area), which represented the single most abundant metabolite. Oligopeptides were also detected in considerable quantities, including an azido-modified peptide (RT 12.71; 8.77 %), a threonyl- and valyl-rich peptide (RT 13.06; 7.91 %), and hydrazine-functionalized peptides (RT 13.45; 6.27 %). Together with a leucine-rich peptide (RT 14.15; 3.50 %), these peptide-derived metabolites accounted for more than 25 % of the total metabolite profile.

**Table 4:** List of non-volatile compounds successfully identified from liquid chromatography-mass spectrometry (LC-MS) analysis.

**No**	**Retention Times**	**Compounds**	**Formulas**	**Component Areas**	**Area (%)**	**Molecular Weight (g/mol)**	**Biocontrol Potentials**	**References**
1	1.28	1-methyl-3-[[methyl(nitroso) carbamoyl] amino]-1-nitrosourea	C_4_H_8_N_6_O_4_	34448	1.59	204.14	Inducing SAR	Klessig et al. (2000)
2	3.06	nitric acid;piperazine	C_4_H_12_N_4_O_6_	6195	0.29	212.16	Lowering the pH of the fungal growth area	Sarangapani et al. (2019)
3	3.85	L-Methionyl-L-isoleucyl-L-histidyl-L-phenylalanylglycyl-L-asparagine	C_32_H_47_N_9_O_8_S	4039	0.19	717.84	Peptides with antifungal potential and plant defense-triggering capabilities	Song et al. (2023); Montesinos et al. (2021)
4	4.57	1,3-dihydroxypropan-2-one;hydrazine;2-hydrazinylidenepropane-1,3-diol	C_6_H_18_N_4_O_5_	2795	0.13	226.23	Hydrazine and hydroxyl groups as phytopathogen inhibitors	Wang et al. (2024); Wang et al. (2020)
5	5.37	1-(1,3-Diazidopropan-2-yl) piperazine	C_7_H_14_N_8_	23038	1.07	210.24	Belongs to the antifungal piperazine compound group	Zhou et al. (2022)
6	5.98	Benzyl (4aS,6aS,6bR,8aR,9R,13aR,13bR,15bS)-2,2,6a,6b,9,13a-hexamethyl-9-({[4-(4-methyl-1-piperazinyl)-4-oxobutanoyl]oxy}methyl)-1,3,4,5,6,6a,6b,7,8,8a,9,13,13a,13b,14,15b-hexadecahydropiceno[2,3-d][1, 2]oxazole-4a(2H)-carboxylate	C_47_H_65_N_3_O_6_	3605	0.17	768.05	Belongs to the triterpenoid and piperazinyl antifungal compound group	Sonkoue et al. (2023); Jalageri et al. (2021)
7	7.43	[2-[2-[2-[2-[2-(2-hydrazinylhydrazinyl)hydrazinyl]hydrazinyl]hydrazinyl]hydrazinyl]hydrazinyl]ethane	C_2_H_20_N_14_	208	0.01	240.28	Includes hydrazinyl antifungal compounds	Zhang et al. (2022); Jaibangyang et al. (2021)
8	8.22	4-[6-amino-5-[(E)-methoxyiminomethyl]pyrimidin-4-yl]-N-[4-(dimethylamino)phenyl]piperazine-1-carboxamide	C_19_H_26_N_8_O_2_	3032	0.14	399.23	Contains piperazine and pyrimidine groups, inducing defense enzymes and antifungal activity	Zhang et al. (2022); An et al. (2023)
9	8.61	Guanidinosuccinic acid	C_5_H_9_N_3_O_4_	478	0.02	175.14	Antifungal and defense induction	Zeng et al. (2025)
10	9.17	N-Acetyl-L-norleucylglycyl-N-(4-aminobutyl)glycyl-D-phenylalanyl-L-ornithyl-L-tryptophylglycinamide	C_43_H_63_N_11_O_8_	3686	0.17	862.05	Potential antifungal peptides and defense inducers	Song et al. (2023); Montesinos et al. (2021)
11	10.77	2-Methyl-2-propanyl (47-hydroxy-3,6,9,12,15,18,21,24,27,30,33,36,39,42,45-pentadecaoxaheptatetracont-1-yl)carbamate	C_37_H_75_NO_18_	56832	2.63	821.99	Carbamate derivatives act as antifungal agents	Al-Sudairy et al. (2024)
12	11.17	(2S)-2-[(2S,3E)-1-{[(1S)-1-Carboxy-2-(4-hydroxyphenyl)ethyl]amino}-1,11-dioxo-3-octadecen-2-yl]-2-hydroxysuccinic acid	C_31_H_45_NO_10_	699950	32.39	591.79	Contains malic acid as a plant defense molecule	Si et al. (2022), Rekha et al. (2018)
13	11.98	26-Azido-N-(26-azido-3,6,9,12,15,18,21,24-octaoxahexacos-1-yl)-3,6,9,12,15,18,21,24-octaoxahexacosan-1-amine	C_36_H_73_N_7_O_16_	8725	0.40	860.01	Contains azido groups for biomolecule conjugation	Lin et al. (2021)
14	12.71	L-Seryl-L-leucyl-L-isoleucylglycyl-L-arginyl-4-azido-L-phenylalanyl-L-isoleucinamide	C_38_H_64_N_14_O_8_	189505	8.77	845.02	Peptides with potential as antifungal agents and plant defense triggers	Song et al. (2023); Montesinos et al. (2021)
15	13.06	L-Valyl-L-prolylglycyl-L-leucyl-L-prolylglycyl-L-threonyl-L-valyl-L-leucine	C_40_H_69_N_9_O_11_	170857	7.91	852.044		
16	13.45	(2R)-1-[(2S)-2-{[(2S,3S)-1-{[(2S)-1-{[(2S)-1-Hydrazino-1-oxo-2-propanyl](methyl)amino}-3-methyl-1-oxo-2-butanyl](methyl)amino}-3-methyl-1-oxo-2-pentanyl]carbamoyl}-1-pyrrolidinyl]-4-methyl-1-oxo-2-pen tanyl 3-aminopropanoate	C_30_H_55_N_7_O_7_	135525	6.27	625.81	Hydrazine derivatives have biocontrol potential	Zhang et al. (2022); Jaibangyang et al. (2021)
17	14.15	2-Methyl-N-octanoylalanylglycyl-L-leucyl-2-methylalanylglycylglycyl-L-leucyl-2-methylalanylglycyl-N-[(3S)-2-hydroxy-5-methyl-3-hexanyl]-L-isoleucinamid	C_53_H_97_N_11_O_12_	75565	3.50	1080.42	Peptides with potential as antifungal agents and plant defense triggers	Song et al. (2023); Montesinos et al. (2021)
18	17.3	N∼2∼,N∼2∼′-1,10-Decanediylbis(1,3,5-triazine-2,4,6-triamine)	C_16_H_30_N_12_	3944	0.18	390.5	Contains triazine with potential for biocontrol	Maliszewski et al. (2023); Yang et al. (2023)

Other notable components included nitrogenous heterocycles and small molecules, such as a carbamate ester (RT 10.77; 2.63 %), a leucine-rich peptide with aliphatic side chains (RT 14.15; 3.50 %), and several piperazine derivatives (RT 5.37 and 8.22; 1.2 % combined). In contrast, guanidinosuccinic acid (RT 8.61; 0.02 %) and polyhydrazinyl compounds (RT 7.43; 0.01 %) were detected at trace levels. The profile also encompassed triazine derivatives (RT 17.3; 0.18 %), azido-substituted molecules (RT 11.98; 0.40 %), and nitrosourea compounds (RT 1.28; 1.59 %), all of which have documented antifungal or plant defense–inducing potential.

Overall, these results highlight that *T. yunnanense* TM10 produces metabolites in varying abundance, with organic acids and peptide-based compounds representing the most quantitatively dominant groups. Given that several of the high-abundance metabolites (e.g., malic acid derivatives, antifungal peptides) are associated with plant defense activation and fungal growth suppression, their relative concentrations support the claim that TM10 functions as a dual biocontrol and growth-promoting agent.

### 3.5 *In vivo* inhibition assay of *T. yunnanense* TM10 against Foc on *A. cepa* var. *aggregatum*

Following the confirmation of *T. yunnanense* TM10’s antagonistic activity against Foc *in vitro*, an *in vivo* assay was conducted. This phase aimed to assess the biocontrol efficacy of *T. yunnanense* TM10 on shallot, a known susceptible host of the pathogen. The evaluation focused on disease suppression under greenhouse conditions, as well as potential plant growth-promoting effects resulting from *T. yunnanense* TM10 application.

#### 3.5.1 Analysis of disease severity index

**Fig. 9:**
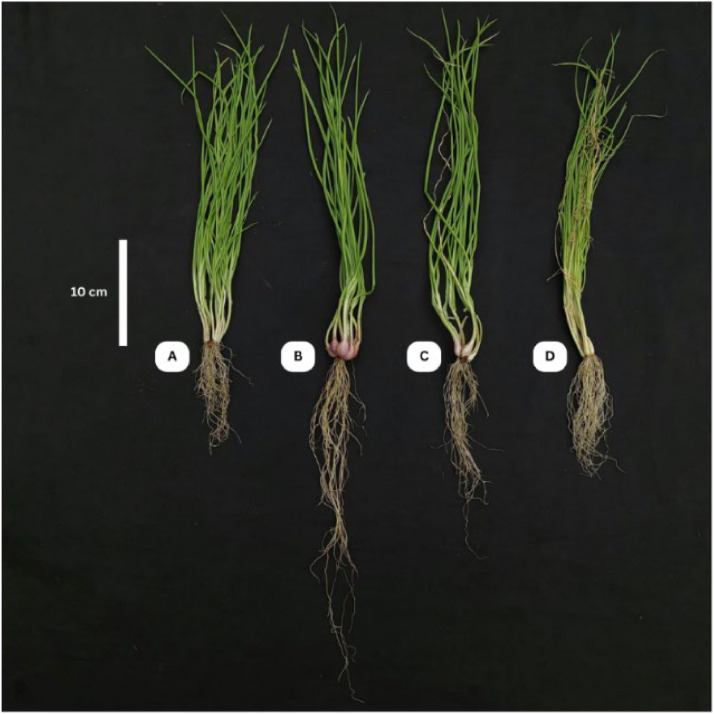
Comparison of *A. cepa* var. *aggregatum* plants under different treatments. (A) Control (untreated); (B) Treated with *T. yunnanense* TM10; (C) Co-inoculated with *T. yunnanense* TM10 and Foc; and (D) Inoculated with Foc.

Initial symptoms of Foc infection on shallot plants were observed at 7 days post-inoculation, characterized by leaf twisting, chlorosis, and wilting. As shown in Fig. 9, Foc-treated plants exhibited stunted growth and yellowed, distorted foliage, in contrast to the vigorous appearance observed in control plants and those treated with *T. yunnanense* TM10.

Quantitative assessment of disease severity using the disease severity index (DSI) revealed significant variation between treatments (Table 5). The Foc-treated plants exhibited the highest DSI value (72.50±3.23 %), indicating severe disease symptoms. In contrast, co-inoculation with *T. yunnanense* TM10 significantly reduced disease severity (33.75±1.25 %), demonstrating its potential biocontrol activity against Foc. These results suggest that *T. yunnanense* TM10 is effective in mitigating *Fusarium*-induced disease in shallot plants under greenhouse conditions.

**Table 5:** Disease severity index values for each treatment.

**Treatments**	**Disease Severity Index (%)**
TM10 + Foc	33.75±1.25^b^
Foc	72.50±3.23^a^

Note: Mean values followed by different lowercase letters within the same column indicate a significant difference according to Student’s *t*-test (*P* < 0.05). Data are presented as mean ± standard deviation.

#### 3.5.2 Analysis of morphological characteristics

Morphological parameters, including root length, leaf length, biomass accumulation, and leaf number, were measured to assess plant growth performance under different treatments. As summarized in Table 6, the *T. yunnanense* TM10 treatment significantly enhanced plant growth compared to all other groups. The longest root length was recorded in the TM10-treated plants (28.45±1.15 cm), while the shortest root length recorded in the Foc-treated plants (10.33±0.62 cm), indicating severe root growth inhibition under pathogen stress. Leaf length followed a similar pattern, with TM10-treated plants reaching 39.58±1.15 cm, outperforming both the control (37.61±3.92 cm) and Foc-treated plants (33.50±3.24 cm).

**Table 6:** Effect of each treatment on shallot morphological growth.

**Treatments**	**Length**		**Weight**		**Number of Leaves**
	**Root (cm)**	**Leaf (cm)**	**Fresh (g)**	**Dry (g)**	
Foc	10.33±0.62^a^	33.50±3.24^a^	9.09±1.08^a^	0.69±0.13^a^	14.95±1.76^a^
Control	13.52±0.78^b^	37.61±3.92^ab^	10.29±0.76^a^	0.86±0.08^ab^	20.35±2.45^b^
TM10 x Foc	20.37±1.43^c^	39.13±1.43^b^	12.67±1.35^b^	1.02±0.05^b^	24.90±2.22^c^
TM10	28.45±1.15^d^	39.58±1.15^b^	17.51±0.80^c^	1.48±0.20^c^	28.65±2.01^d^

Note: Mean values followed by the same lowercase letter within the same column indicate no significant difference according to DMRT test (*P* < 0.05). Data are presented as mean ± standard deviation.

Biomass accumulation was also highest in the TM10 treatment, with fresh and dry weights of 17.51±0.80 g and 1.48±0.20 g, respectively. The TM10 + Foc combination showed improved growth relative to the Foc-treated plants, though slightly lower than TM10-treated plants. Leaf number was markedly increased in the TM10-treated plants (28.65±2.01), compared to 20.35±2.45 in the control and only 14.95±1.76 under Foc-treated plants. These findings indicate that *T. yunnanense* TM10 not only mitigates the detrimental effects of Foc, but also promotes vegetative growth and biomass production of shallot supporting its dual function as a biocontrol and plant growth-promoting agent.

#### 3.5.3 Analysis of physiological characteristics

To evaluate the physiological responses of shallot plants to biocontrol treatment and pathogen challenge, a series of physiological parameters were assessed. These included chlorophyll content, total phenolic compounds, and the activity of key defense-related enzymes, namely catalase (CAT) and peroxidase (POD).

##### 3.5.3.1 Effect of *T. yunnanense* TM10 inoculation on chlorophyll levels in shallot plants

Chlorophyll content served as a physiological indicator of plant health under biocontrol and pathogen treatments. As shown in Table 7, the lowest levels of chlorophyll *a* (1.27±0.0012 mg/g), chlorophyll *b* (0.19±0.002 mg/g), total chlorophyll (1.46±0.001 mg/g), and carotenoids (0.51±0.001 mg/g) were recorded in the Foc-treated plants, indicating pronounced stress. In contrast, the TM10-treated plants showed the highest pigment concentrations, with chlorophyll *a* at 2.20±0.0016 mg/g, chlorophyll *b* at 0.82±0.002 mg/g, total chlorophyll at 3.02±0.002 mg/g, and carotenoids at 0.83±0.005 mg/g. The TM10 + Foc treatment also resulted in elevated chlorophyll *a* (2.11±0.0018 mg/g), total chlorophyll (2.46±0.004 mg/g), and carotenoids (0.84±0.001 mg/g), surpassing both the control and Foc-treated plants.

**Table 7:** Comparison of chlorophyll content analysis results for each treatment.

**Treatments**	**Chlorophyll *a* (mg/g)**	**Chlorophyll *b* (mg/g)**	**Total chlorophyll (mg/g)**	**Carotenoids (mg/g)**
Control	1.40±0.0017^b^	0.51±0.002^c^	1.92±0.001^b^	0.74±0.001^b^
TM10	2.20±0.0016^d^	0.82±0.002^d^	3.02±0.002^d^	0.83±0.005^c^
TM10 + Foc	2.11±0.0018^c^	0.35±0.006^b^	2.46±0.004^c^	0.84±0.001^d^
Foc	1.27±0.0012^a^	0.19±0.002^a^	1.46±0.001^a^	0.51±0.001^a^

Note: Mean values followed by the same lowercase letter within the same column indicate no significant difference according to DMRT test (*P* < 0.05). Data are presented as mean ± standard deviation.

Based on the analysis, the application of *T. yunnanense* TM10 not only protects shallot plants from the degradation of photosynthetic pigments caused by Foc infection but also actively enhances their photosynthetic capacity. These findings demonstrate TM10’s dual role in mitigating pathogen-induced physiological stress and promoting the accumulation of key photosynthetic compounds.

##### 3.5.3.2 Effect of *T. yunnanense* TM10 inoculation on total phenol content in shallot plant

Total phenolic content was measured as an indicator of induced defense responses in shallot plants. As shown in Table 8, the highest phenol accumulation was recorded in the TM10 + Foc treatment (704.59±0.26 mg/L), significantly surpassing all other treatments. Foc-treated plants exhibited increased phenol levels (567.91±0.96 mg/L) relative to the control (423.16±1.42 mg/L) and TM10-treated plants (455.12±0.81 mg/L), indicating a stress-induced response to pathogen attack.

**Table 8:** Comparison of defense enzyme analysis results for each treatment.

**Treatments**	**Total phenolic content (mg/L)**
Control	423.16±1.42^a^
TM10	455.12±0.81^b^
TM10 + Foc	704.59±0.26^d^
Foc	567.91±0.96^c^

Note: Mean values followed by the same lowercase letter within the same column indicate no significant difference according to DMRT test (*P* < 0.05). Data are presented as mean ± standard deviation.

The marked elevation in phenolic content in the TM10 + Foc group suggests that *T. yunnanense* TM10 enhances phenolic-based defense mechanisms beyond the plant’s innate response. This result highlights TM10’s potential role as an effective inducer of systemic resistance, contributing to its biocontrol efficacy through the stimulation of secondary metabolite pathways.

##### 3.5.3.3 Effect of *T. yunnanense* TM10 inoculation on defense enzymes activity in shallot plants

Defense-related enzyme activity was evaluated to assess the physiological response of shallot plants to *T. yunnanense* TM10 application under Foc challenge. As shown in Table 9, catalase (CAT) and peroxidase (POD) activities were lowest in the control treatment (211.18±0.75 U/L and 3,537.39±0.06 U/L, respectively), reflecting basal enzyme levels. Application of TM10-treated plants increased CAT and POD activities to 354.43±1.76 U/L and 4,641.87±0.14 U/L, indicating defense activation even in the absence of pathogen stress.

**Table 9:** Comparison of defense enzymes analysis results for each treatment.

**Treatments**	**CAT activity (U/L)**	**POD activity (U/L)**
Control	211.18±0.75^a^	3,537.39±0.06^a^
TM10	354.43±0.1.76^b^	4,641.87±0.14^b^
TM10 + Foc	686.11±0.74^d^	5,947.76±0.51^c^
Foc	599.76±0.90^c^	4,095.77±0.03^ab^

Note: Mean values followed by the same lowercase letter within the same column indicate no significant difference according to DMRT test (*P* < 0.05). Data are presented as mean ± standard deviation.

The TM10 + Foc treatment yielded the highest enzyme activities, with CAT at 686.11±0.74 U/L and POD at 5947.76±0.51 U/L, demonstrating a robust induced systemic resistance (ISR) response. Although Foc-treated plants also elevated enzyme levels (599.76±0.90 U/L for CAT and 4,095.77±0.03 U/L for POD), these values remained significantly lower than those observed in the TM10 + Foc treatment. These results indicate that *T. yunnanense* TM10 enhances the enzymatic defense capacity of shallot plants, particularly under pathogen pressure, likely contributing to improved oxidative stress management and cellular reinforcement.

## 4 Discussions

*T. yunnanense* TM10 exhibits potential as a dual-function biological agent, capable of suppressing disease and promoting growth in shallots challenged by Foc. *In vitro* assays revealed strong antagonistic interaction, where dual culture assays demonstrated significant antagonistic activity, with TM10 achieving the highest inhibition rate against Foc, highlighting its strong competitiveness for space and nutrients (Table 1) (Yao et al., 2023; Guzmán-Guzmán et al., 2023). Despite being inoculated two days after Foc, TM10 rapidly overgrew and dominated the Petri dish by day five, demonstrating strong competitive ability for space and nutrients (Yao et al., 2023; Guzmán-Guzmán et al., 2023). Microscopic observation via slide culture confirmed mycoparasitic activity, notably hyphal coiling, an early-stage antagonistic behavior widely observed in *Trichoderma* spp. (Tyśkiewicz et al., 2022; Mukherjee et al., 2022). Sehim et al. (2023) similarly reported that *T. asperellum* inhibited *F. oxysporum* through coiling and hyphal degradation.

The dual culture assay efficacy of *T. yunnanense* TM10 was notably higher than that reported for several other *Trichoderma* species. Previous studies have shown inhibition rates of 68–72 % with *Trichoderma* spp. (Poromarto et al., 2022; Moka et al., 2021) and 61–73 % with *T. harzianum* (Coşkuntuna and Özer, 2008; Hersanti et al., 2020). By comparison, TM10 demonstrated superior antagonistic performance, positioning it as a highly effective candidate for the biocontrol of Foc in shallot cultivation.

The culture filtrate assay reflected the effect of non-volatile diffusible compounds and showed increased activity as the filtrate aged (Table 1, Table 2). This trend supports findings by Boukaew et al. (2024), who observed higher inhibition with older filtrates of *T. asperelloides* SKRU 01. The efficacy observed in this study was notably higher than that reported for *T. harzianum, T. viride*, and *T. haematum*, which showed inhibition levels of only 5–20 % (Ghanbarzadeh et al., 2014). For volatile compounds assay, TM10 outperformed previous reports on *T. haematum* (15–20 %), *T. harzianum* (1–15 %), and *T. viride* (15–20 %) against Foc (Ghanbarzadeh et al., 2014). Although the inhibition rate from volatile metabolites was lower than that of culture filtrates, it still exceeded values reported for other *Trichoderma* species, underscoring the role of antibiosis.

When compared with chemical fungicides, TM10’s antibiosis effect was weaker but still considerable. Degani and Kalman (2021) reported that, after a 6-day incubation period, compounds such as thiophanate-methyl, carbendazim, pyrimethanil, fludioxonil, azoxystrobin, prochloraz, tebuconazole, pyraclostrobin-boscalid, fludioxonil-sedaxane, and azoxystrobin-tebuconazole suppressed Foc growth, resulting in colony diameters of approximately ±0.5 to ±3.5 cm. In comparison, TM10 exhibited inhibition values of 4.11 cm in the culture filtrate assay and 5.41 cm in the volatile compound assay, indicating a relatively weaker but still substantial antifungal capacity. Importantly, unlike synthetic fungicides that may pose risks of environmental accumulation, resistance development, and non-target toxicity, fungal strains of those from *Trichoderma* genus such as TM10 represent an eco-friendly and sustainable alternative for disease management (Feng et al., 2020; Gikas et al., 2022; Guo et al., 2021; Thambugala et al., 2020; Palmieri et al., 2022). Therefore, TM10 offers a safer and more sustainable approach for long-term shallot cultivation compared to reliance on chemical fungicides.

GC-MS analysis confirmed the presence of bioactive volatiles such as ethanol, cyclotetrasiloxane octamethyl, and butylated hydroxytoluene, representing alcohol, siloxane, and substituted phenol classes respectively, all of which are known for their antifungal properties (Table 3) (Steglińska et al., 2022; Joo and Husein, 2022; Sebestyen et al., 2022; Oviya et al., 2022). Additionally, LC-MS profiling revealed a diverse array of nonvolatile secondary metabolites (Table 4). Five abundant and structurally identified compounds, including an organic acid derivative, oligopeptide complexes, a hydrazine-functionalized peptide, and a carbamate compound, represented major chemical classes commonly associated with antifungal and defense-inducing activity (Si et al., 2022; Song et al., 2023; Zhang et al., 2022; Al-Sudairy et al., 2024). Other detected classes included triazine derivatives, piperazine-based heterocycles, polyhydrazinyl compounds, and nitrogen-containing small molecules such as guanidinosuccinic acid and nitrosourea analogs. This chemical diversity highlights the complexity of TM10′s secondary metabolome and its potential synergistic roles in biocontrol.

*In vivo* greenhouse trials confirmed the biocontrol efficacy of *T. yunnanense* TM10, with the highest DSI observed in the Foc-treated plants (Table 5). Characteristic symptoms such as foliar wilting, chlorosis, and growth suppression were evident, reflecting the virulence of Foc, which disrupts vascular function and impairs water and nutrient transport (Awal et al., 2024; Srinivas et al., 2019). In contrast, DSI in the TM10 + Foc treatment decreased by more than 50 %, indicating effective suppression, likely due to the production of hydrolytic enzymes, antifungal volatiles, and the activation of systemic resistance (Panchalingam et al., 2022; Hung et al., 2013).

Similiarly, plant morphological observations further support the growth-promoting potential of TM10 (Table 6). TM10-treated plants exhibited the most vigorous growth, as reflected in root and leaf elongation, increased biomass, and greater leaf number. In contrast, Foc-treated plants showed significant growth inhibition, consistent with typical pathogen-induced stress responses (Sakane et al., 2023; Dutta et al., 2022). The TM10 + Foc treatment resulted in improved growth compared to Foc-treated plants, indicating that TM10 effectively alleviates disease-related growth suppression. Previous studies using *Trichoderma* in *Allium* crops reported similar enhancements, attributed to disease suppression, hormone production, and improved nutrient mobilization (Abdelrahman et al., 2016; Rodríguez-Hernández et al., 2023).

Physiological analyses revealed that TM10 treatments improved photosynthetic pigment content (Table 7). Infection by Foc is known to impair chloroplast integrity and trigger pigment degradation, likely mediated by fusaric acid toxicity (Sharma and Sharma, 2020; Perincherry et al., 2019). In contrast, *T. yunnanense* TM10 appeared to enhance chlorophyll and carotenoid content, potentially through improved nutrient availability and presence of butanal, 3-methyl- (isovaleraldehyde), which has been associated with hormone signaling and chlorophyll biosynthesis (Jiang et al., 2021; Hung et al., 2013). Hung et al. (2013) further demonstrated this compound's role in increasing chlorophyll content in *Arabidopsis thaliana*. Notably, the TM10 + Foc treatment maintained higher pigment levels than the Foc-treated plants, indicating TM10′s protective effect on photosynthetic function (Abdelrahman et al., 2016; Rodríguez-Hernández et al., 2023).

Beyond photosynthetic protection, TM10 also enhanced biochemical defense mechanisms (Table 8, Table 9). Total phenolic content, a key marker of stress-related defense responses, was highest in plants treated with both TM10 and Foc, indicating that TM10 may enhance phenolic biosynthesis under pathogen challenge. This pattern suggests the activation of ISR, where TM10 primes the host plant’s defense machinery prior to or during infection (Attia et al., 2025). Similar increases in phenol levels were reported following *Trichoderma* applications in onion and shallot by Abdelrahman et al. (2016) and Rodríguez-Hernández et al. (2023), reinforcing TM10’s role in activating chemical defenses (Mironenka et al., 2021).

The TM10 treatment elevated CAT and POD activities, which are essential components of the plant’s enzymatic antioxidant defense system (Table 9). The treatment combining TM10 with Foc resulted in the highest levels of both enzymes, indicating a strong activation of induced systemic resistance. Although Foc-treated plants also led to increased CAT and POD activity, the levels were notably lower than those observed in plants treated with TM10, emphasizing TM10’s role in priming and enhancing oxidative stress responses under pathogenic conditions (Poursakhi et al., 2025; Mishra et al., 2023; dos Santos and Octávio, 2023).

Although this study highlights the strong potential of *T. yunnanense* TM10 under greenhouse conditions, the results should be viewed in light of certain limitations. Field environments are far more complex than controlled settings, with fluctuating temperatures, diverse soil microbiomes, and variable nutrient conditions that may influence *T. yunnanense* TM10’s consistency. Moving from greenhouse trials to field application also brings practical challenges, such as developing stable formulations, suitable delivery methods, and ensuring the inoculant persists in the rhizosphere over time. Further studies are therefore needed to evaluate *T. yunnanense* TM10’s performance under different field conditions and to refine strategies for its integration into broader disease management programs.

## 5 Conclusion

*T. yunnanense* TM10 demonstrates strong potential as a dual-function biological agent for managing Foc as well as promoting growth in shallots. It effectively suppressed pathogen growth through multiple mechanisms, including mycoparasitism, competition, and antibiosis, supported by the production of diverse volatile and nonvolatile antifungal metabolites. *In vivo*, TM10 reduced disease severity by over 50 %, preserved photosynthetic pigment levels, and significantly enhanced phenolic content, antioxidant enzyme activity, and overall plant growth under pathogen infection. These results highlight *T. yunnanense* TM10’s capacity to not only control disease but also promote plant vigor, positioning it as a promising candidate for integrated biocontrol and growth enhancement strategies in shallot cultivation. Future studies should focus on validating its efficacy under field conditions, optimizing formulation and delivery methods, and exploring its broader applicability across different crops and agroecological systems.

## Funding

This work was funded by Universitas Padjadjaran through Riset Keunggulan Keilmuan Unpad (RKKU) (Contract Number: 950/UN6.3.1/PT.00/2025) awarded to Febri Doni.

## Declaration of competing interest

The authors declare that they have no known competing financial interests or personal relationships that could have appeared to influence the work reported in this paper.
